# Optimization of Chaboche Material Parameters with a Genetic Algorithm

**DOI:** 10.3390/ma16051821

**Published:** 2023-02-22

**Authors:** Nejc Dvoršek, Iztok Stopeinig, Simon Klančnik

**Affiliations:** 1 Faculty of Mechanical Engineering, University of Maribor, Smetanova 17, 2000 Maribor, Slovenia; 2 AVL-AST d.o.o., Ulica Kneza Koclja 22, 2000 Maribor, Slovenia

**Keywords:** Chaboche material model, parameter optimization, genetic algorithm, finite element method

## Abstract

The main objective of this study is to research and develop a genetic algorithm (GA) for optimizing Chaboche material model parameters within an industrial environment. The optimization is based on 12 experiments (tensile, low-cycle fatigue, and creep) that are performed on the material, and corresponding finite element models were created using Abaqus. Comparing experimental and simulation data is the objective function that the GA is minimizing. The GA’s fitness function makes use of a similarity measure algorithm to compare the results. Chromosome genes are represented with real-valued numbers within defined limits. The performance of the developed GA was evaluated using different population sizes, mutation probabilities, and crossover operators. The results show that the population size had the most significant impact on the performance of the GA. With a population size of 150, a mutation probability of 0.1, and two-point crossover, the GA was able to find a suitable global minimum. Comparing it to the classic trial and error approach, the GA improves the fitness score by 40%. It can deliver better results in a shorter time and offer a high degree of automation not present in the trial and error approach. Additionally, the algorithm is implemented in Python to minimize the overall cost and ensure its upgradability in the future.

## 1. Introduction

Some real-world materials and their behavior are complex and hard to model. Such materials are usually used in dynamic simulations with components that are stressed beyond their elastic limits. Fatigue simulations are one of them; dealing with high temperatures introduces creep, and cyclic loading affects materials’ hardening or softening. A material that can describe such complex behavior is called Chaboche, a nonlinear kinematic hardening model [1,2].

The Chaboche material model is a commonly used model for simulating the behavior of stainless steels, aluminum, nickel, and copper alloys [3,4,5,6,7]. However, it is characterized by a set of parameters that cannot be measured directly from experimental data, such as the Young’s modulus. Additionally, the relationships between these parameters are nonlinear and unknown. In order to use this model in simulations accurately, it is necessary to determine its parameters through an inverse analysis. One approach that has been successful in this regard is the use of a genetic algorithm (GA).

The GA tries to imitate Darwinian evolution that is seen in nature to improve a population of individuals over generations and converge to a global minimum. The GA can work with unknown functions, if it is possible to score each individual in a way that is meaningful to the optimization problem. GA has proved useful for optimizing material design and parameters. Components can be made from different material properties in different regions, depending on the requirements for its application. In the study completed by X.-J. Zhang and others, they proposed a method with improved GA to optimize the material properties in each region of the component [8]. E. O. Vazquez and others demonstrated that GA can be utilized to optimize the material design of one-dimensional photonic crystal mirrors made of porous silicon [9]. GAs are a well-established method of optimization in various fields, for example: robotics, economics, automated design scheduling tasks, vehicle routing, and more [10,11,12,13].

It has been demonstrated that GAs can address the challenge of extracting the Chaboche model’s parameters successfully from experimental data. A custom software for optimizing the parameters of 42CrMo4 steel was created by M. Franulovi and colleagues [14]. A multi-objective genetic algorithm (MOGA) was used by A. H. Mahmoudi and others to enhance ratcheting prediction and characterize the material hysteresis loop precisely [15]. In their research to optimize Chaboche parameters for SS304 stainless steel, H. Badnava and colleagues also used MOGA [16]. D. Agius and others performed a thorough sensitivity study for the MOGA-II algorithm using modeFRONTIER commercial software [17]. For stainless steel 316L material parameters, N. Moslemi compared GA with a related particle swarm optimization approach [18]. S. Mal optimized the 20MnMoNi55 steel parameters using MATLAB’s sequential GA [19].

The scope of this work is to develop a GA for material parameter identification within an industrial setting and doing so without any additional commercial software, except the Abaqus finite-element method solver. To assess its performance, a parameter study w conducted on the algorithm. The developed GA was compared against a classic trial and error optimization approach, highlighting the differences between them. This work constitutes a novel contribution in that it implements a GA for material parameter identification in an industrial environment, which brings forth additional challenges such as limited computational resources and time constraints. For these reasons the algorithm must be efficient and flexible, enabling quick implementation for the problem at hand. If the GA can achieve similar or superior results compared to the trial and error approach while facilitating a more automated workflow, this would signify the success of using the GA for optimization.

## 2. Materials and Methods

### 2.1. Data

**Figure 1:**
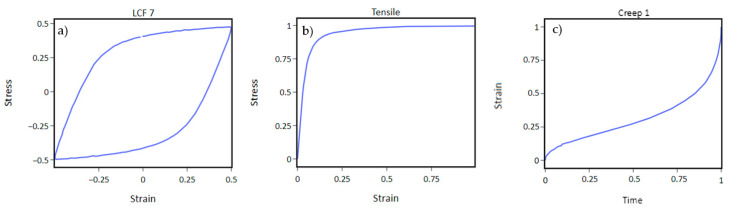
LCF (**a**), tensile (**b**), and creep (**c**) experimental data used for the optimization.

The data from 12 experiments are available for optimization. They are tensile, seven different low-cycle fatigue (LCF), and four different creep tests (Figure 1). Only one stabilized hysteresis loop is used from the LCF experiments.

The study is completed in collaboration with AVL-AST d.o.o. (AVL) who is providing the experimental data, Abaqus FEM models, licenses, and computer resources. The raw material data are confidential and cannot be shared; therefore, all figures containing material data are presented with normalized values from 0 to 1. Normalizing values is not a bug, but a feature necessary to ensure experiments are comparable with each other. For example, stress and strain values are different in orders of magnitude and otherwise not comparable. In the process, normalization of values is introduced before scoring an individual. Normalized data from that point forward are shown throughout the paper.

### 2.2. Manual Fitting

Optimization for the available material data is first completed with AVLs experts with a trial and error method [20], allowing exploration of the design space without the risk of ignoring the influence of any parameters. The final step of fine-tuning the parameter values is carried out manually based on the expert’s experience. This is the current method used by AVL and is called manual fitting (MF) throughout the article. The results from this true and tested approach serve as a reference to the developed GA.

### 2.3. Simulation Model

**Figure 2:**
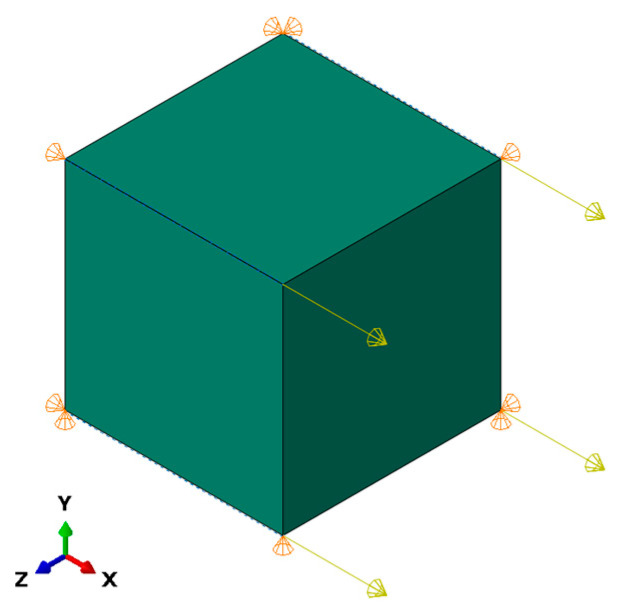
Abaqus FEM model. C3D8 element with load applied in the *X* direction and fixed on the other side in a way that introduces uniaxial loading scenario.

For every experiment, there is an equivalent Abaqus finite element method (FEM) model available that describes it accurately. The duration of simulations impacts the optimization time greatly; therefore, only one quadratic (C3D8) element was used (Figure 2). Loads and boundary conditions are taken directly from the experiments themselves. For example, in the case of the tensile experiment, displacement from experimental data is used as a boundary condition for the simulation. The load is applied on one side to all four nodes in the positive *X* direction. Boundary conditions are applied in a way that uniaxial stress state is achieved. Namely displacements perpendicular to loading directions are free, hence no stress in those directions. With this assumption a single element is sufficient for capturing the desired behavior. A side effect of this decision are fast simulation times.

### 2.4. Chaboche Material Model

A formulation from Abaqus manual is used for describing the material model [21]. The yield surface used is the von Mises surface. Where s is the deviatoric stress tensor and a is the deviatoric part of the backstress tensor.

(1) $$f\left(\sigma -a\right)=\sqrt{\frac{3}{2}\left(s-a\right):\left(s-a\right)}$$

Strain rate can increase yield strength and must be taken into account. The following equation describes the plastic strain rate. ${\dot{\varepsilon }}_{0}$, K, and n are material constants that must be optimized.

(2) $${\dot{\bar{\varepsilon }}}^{pl}={\dot{\varepsilon }}_{0}{\langle \frac{\bar{\sigma }-\sigma^{0}}{K}\rangle }^{n}$$

The kinematic hardening component is defined to be an additive combination of a purely kinematic term (linear Ziegler hardening law), a relaxation term (the recall or dynamic recovery term), and, optionally, a static recovery term. The last two terms introduce the nonlinearity. In addition, several kinematic hardening components (backstresses) can be superposed, which may considerably improve the results in some cases. When temperature and field variable dependencies are omitted, the hardening laws for each backstress are [21]:

(3) $$\dot{\alpha_{k}}=C_{k}\cdot \frac{1}{\sigma_{0}}\left(\sigma -\alpha \right){\dot{\bar{\varepsilon }}}^{pl}-\gamma_{k}\cdot {\dot{\bar{\varepsilon }}}^{pl}\cdot \alpha_{i}-\xi_{k}\cdot {\left(\frac{\left|\alpha_{k}\right|}{R_{k}}\right)}^{m_{k}-1}\cdot \alpha_{k}$$

and the overall backstress is computed from the relation:

(4) $$\alpha =\overset{N}{\underset{k=1}{\sum }}\alpha_{k}$$

where N is the number of backstresses, and $C_{k}$, $\gamma_{k}$, $\xi_{k}$, $R_{k}$ and $m_{k}$ are material parameters that must be calibrated from cyclic test data. $C_{k}$ is the initial kinematic hardening moduli, and $\gamma_{k}$ determines the rate at which the kinematic hardening occurs.

The isotropic hardening of the material is defined by $R_{e}$ yield stress at zero plastic strain and $Q_{\infty }$ and b which are material parameters.

(5) $$\sigma^{0}=R_{e}+Q_{\infty }\left(1-e^{-b{\bar{\varepsilon }}^{pl}}\right)$$

A more detailed description of the Chaboche material model can be found in a book from J. Lemaitre and J.L. Chaboche [1] that describes elastoplasticity and related material behaviors in great detail.

The Chaboche material model configuration must be the same as the given reference from the MF method to ensure a fair comparison between the two approaches. Isotropic hardening is not needed, since the experimental data available are for only one cycle and there was no hardening/softening behavior. From Equation (5), only constant $R_{e}$ is used. Two kinematic hardening components were considered (Equation (3)). The second one did not use the static recovery part of kinematic hardening. This means that, out of two kinematic hardening components, one does not have the material parameters $\xi_{k}$, $R_{k}$ and $m_{k}$. From Equation (2) that defines the plastic strain rate, three additional material parameters ${\dot{\varepsilon }}_{0}$, K and n must be considered. Altogether the material model was described by 11 parameters. To limit the parameter space further, the limits and step sizes for each parameter are set by a material expert at AVL (Table 1).

### 2.5. Genetic Algorithm

What is known about the material is how it behaves from experimental tests. Simulating these tests with FEM and comparing them with actual experimental data is the objective function by which individuals are scored in a population. Minimizing this function is the optimization problem the GA is used for. The GA is imported through the Pygad Python library [22]. All the basic concepts of GAs are available in this library. Other libraries used are Numpy [23] for array manipulation and Similaritymeasures to calculate the area between the two curves, proposed in this paper by [24]. A real-valued number representation was used for the chromosome genes (Table 2). How operators used by GA work can be found in Pygad documentation [22].

The fitness function works in the following steps:

1. Replace the old parameters in the Abaqus input file with new ones;
2. Submit all 12 Abaqus jobs in the background;
3. Wait for all jobs to finish;
4. Read the Abaqus.odb file and write *XY* data (e.g., stress—strain and strain—time) to a separate text file;
5. Obtain the similarity measure (SM) between the experiment and simulation data;
6. Sum all 12 SMs and inverse them to obtain the fitness score

**Figure 3:**
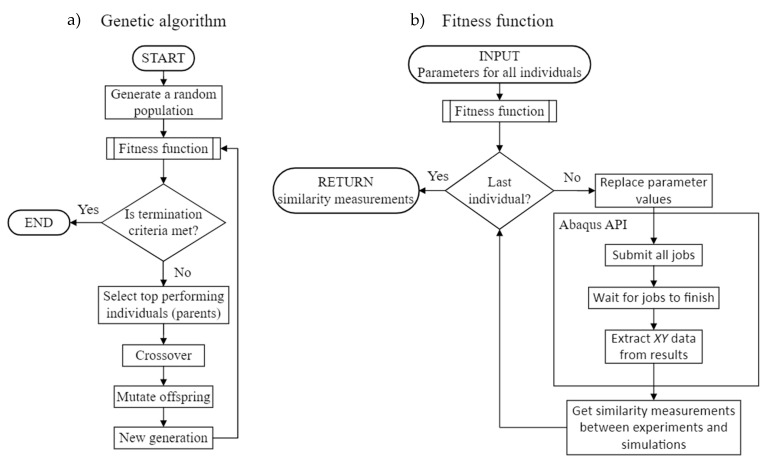
Optimization process flow diagram. GA (**a**) calculates the fitness score with a subprogram (**b**). The subprogram manages all Abaqus simulations and extracts results from them. Results are returned to the GA. Best performing individuals are selected as parents through crossover and mutation operations a new population is formed.

Changing old material model parameters with new ones works by finding and replacing the required strings in a text file. Not every job is submitted or finished at the same time. Fortunately, Abaqus writes a status file with each job, where it can be checked if the simulation is finished or not. This is checked every second, and when all simulations are completed, the program continues. The simulation results are written in an output database (.odb) file that is proprietary to Abaqus and can only be read with Abaqus API. Exporting the *XY* data to a text file is crucial, so that the main process can access it and manipulate it further on. The optimization process flow chart is shown in Figure 3.

**Figure 4:**
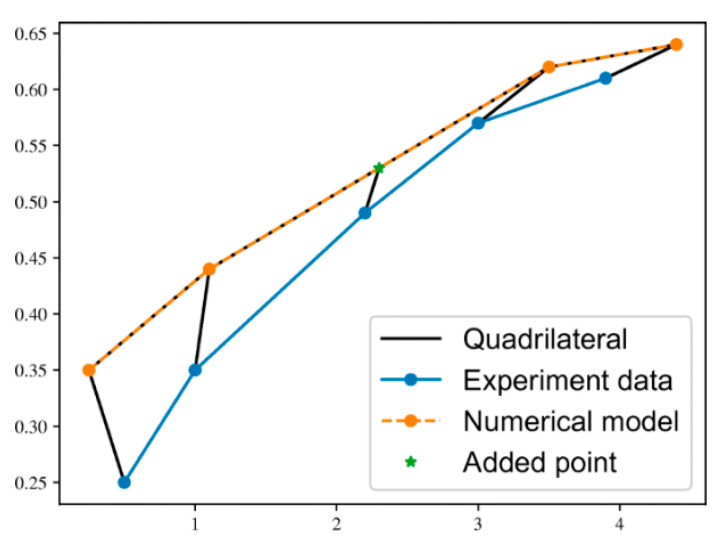
Example of the area calculation method [24]. It starts by ensuring both datasets are the same size, by adding points to the curve smaller in size (green added point). Connecting curve points in order forms quadrilaterals. The area of all quadrilaterals is calculated and summed together.

A real-valued function known as SM quantifies how similar two objects are to one another. In our case, measuring the similarity between two curves in a 2D space. The algorithm used was proposed in this paper by C. F. Jekel and others [24] (Figure 4). Two curves that are being compared require the same number of points in order to construct quadrilaterals to approximate the total area between the curves. If one curve has fewer data points than the other curve, data points are added until both curves have the same number of data points. It was chosen to add rather than remove points to avoid any loss of information for general problems. While there are many ways to add artificial data points to the curve, a simple approach was taken which adds an artificial data point by bisecting two points. The location for the bisection was chosen based on the largest Euclidean distance between two consecutive points. Points are added to the curve in this fashion until both curves have the same number of points [24].

To obtain the final fitness score for an individual, SMs for all experiments must be calculated and summed together and inverted because the Pygad library expects a higher number to represent a better solution. Normalized experimental data ensure that all experiments are equally important, and there is no bias towards any particular one.

(6) $$Fitness\;score={\left(\overset{12}{\underset{i=1}{\sum }}SM_{i}\right)}^{-1}$$

### 2.6. Parallization

After initial testing, it was rapidly obvious that the fitness function cannot run in a sequential manner, since it takes too much time to obtain any meaningful results. To finish all 12 simulations and calculate a fitness score for one individual takes 36 s on an available 40 core workstation with 2.4 GHz core clock speeds.

With parallelization of the fitness function added, the GA can run fitness score evaluations for the whole population at the same time. However, with CPU power limited to 40 cores, only 3 individuals can be evaluated at the same time. To best utilize the hardware, the population size must be divisible by three. After the GA has fitness scores for each individual, it runs sequentially to create a new generation. Parallelization plays a crucial part in the practicality of the algorithm.

## 3. Results and Discussion

First, a general robustness and accuracy analysis based on benchmarking functions for GAs [25] is performed. These functions take into account the effect of population size, crossover probability, mutation rate, and pseudorandom generator. Next, a parameter sensitivity study is performed to test the performance of the developed GA on the experimental data. Initial testing was completed with a population size of 18 individuals. The initial guess was that the GA needs more than 100 generations to find an optimum; therefore, it is not possible to use a big population because of the time it takes to finish the optimization. These tests did not yield promising results. The GA become stuck in a local minimum. Giving up on the initial guess proved that a bigger population size is a key factor to finding an optimal solution.

After this breakthrough further testing was carried out to see how different parameters impacted the GA’s performance. The population size, mutation probability, and crossover operators were varied in the parameter study. The parent selection method used was steady state and held constant. It introduced a strong survival criterion, meaning that only a number of the fittest individuals (20) were selected, and others did not have a chance of reproducing.

### 3.1. Population Size

Parameters:

- Population size = 18, 99, 150, 198
- Crossover = Two-point crossover
- Mutation probability = 0.1

**Figure 5:**
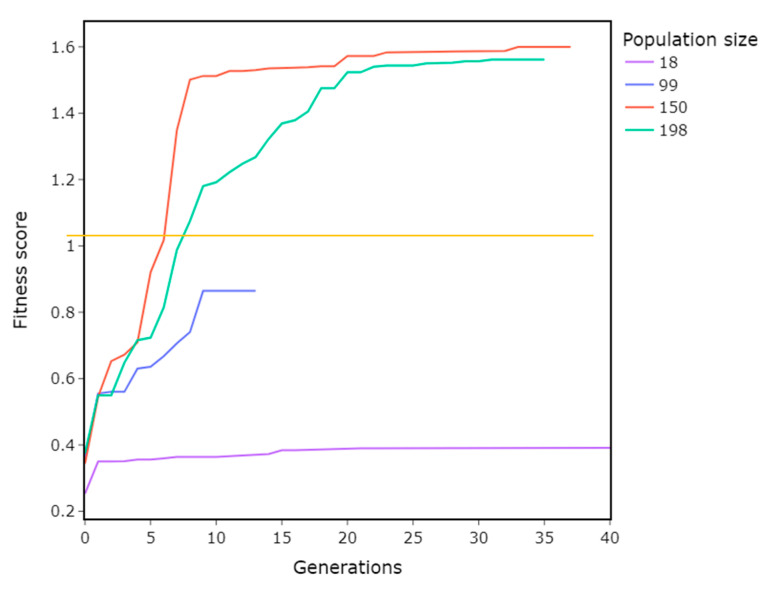
Impact of population size on the GA’s performance. Higher population sizes show better performance in terms of the fitness score. The population size of 150 shows the best results.

The bigger population size means that the GA has more points in each generation available for optimization, enabling it to explore more of the parameter space and converge faster. This can be seen in Figure 5, where there is a big difference in performance between the population sizes 18 and 150. The reference parameters from the MF method obtained a fitness score of 1.05, represented by the yellow horizontal line in Figure 5. A population size of 99 did not break this threshold. Meanwhile, a size of 150 broke this threshold, and even improved it by 40% compared to the MF method. The difference between 150 and 198 is minimal, and suggests diminishing returns with even higher population sizes.

### 3.2. Mutation Probabiilty

Parameters:

- Population size = 150
- Crossover = Two-point crossover
- Mutation probability = 0.1, 0.1, 0.2

**Figure 6:**
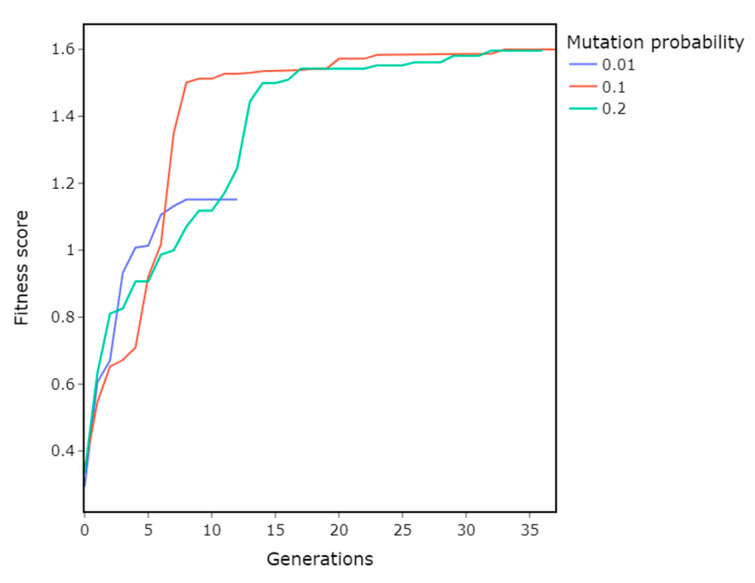
Impact of mutation probability on the GA’s performance. The mutation probability of 0.1 shows the best performance. A higher probability of 0.2 does not perform better and a lower probability of 0.01 underperforms.

Mutation probability contributes to parameter space exploration. Too low a probability and the GA becomes stuck in a local minimum, too big and it is not able to narrow down on the optimum. Out of the tested mutation probabilities, 0.01 performed the worst; a higher value of 0.1 yielded better results. Further increasing the probability to 0.2 did not have any significant impact on performance (Figure 6).

### 3.3. Crossover Operator

Parameters:

- Population size = 150
- Crossover = Two-point and single-point
- Mutation probability = 0.1

**Figure 7:**
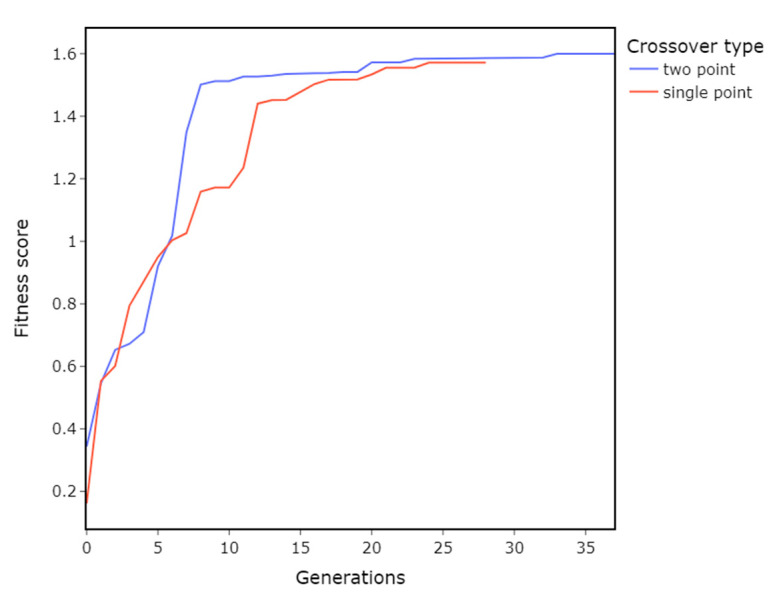
Impact of crossover operators on the GA’s performance. There is no significant difference between the two- and single-point crossovers.

The main difference between single and two points crossover is that two points can explore the parameter space faster at the cost of breaking up more of the genetic sequence. For some problems, disrupting genetic sequence too much can impact the GA’s performance. However, in this case, two point- and single-point operators performed similarly (Figure 7).

### 3.4. Discussion

**Figure 8:**
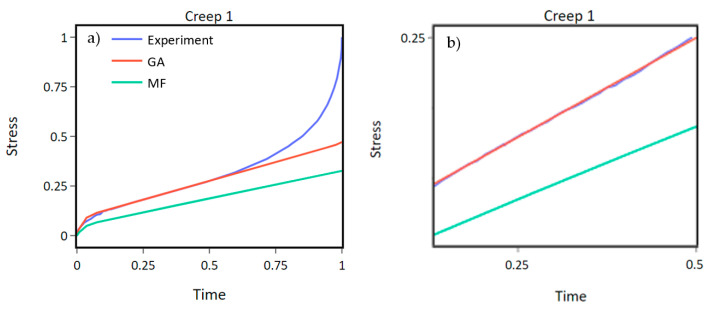
GA and MF methods compared to the experimental creep 1 curve. (**a**) shows the whole creep curve. (**b**) is focused on the linear part of the curve. The GA finds a better fit than the MF method.

**Figure 9:**
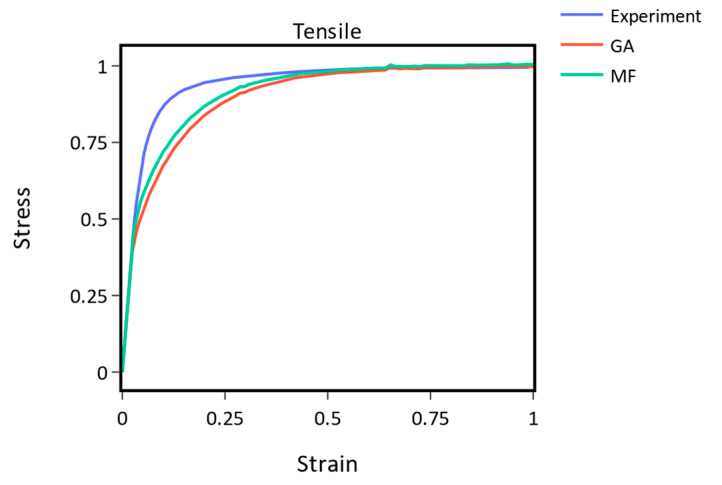
The GA and MF methods compared to the experimental tensile curve. The MF method outperforms the GA.

Creep 1 (Figure 8a) and the tensile experiment (Figure 9) demonstrate the best and worst performing cases when comparing the GA and MF methods.

The secondary linear portion of the experimental creep curve, as illustrated in Figure 8b, is utilized in the optimization process as the Chaboche material model is inadequate in describing both the primary and tertiary portions. In creep experiment 1, the GA effectively generated a set of optimal parameters that accurately fit the curve, whereas the MF method lagged behind in its solution.

Conversely, for the tensile experiment depicted in Figure 9, the MF method outperformed the GA by a margin of 13% as indicated in Table 3. It is worth noting that a perfect fit was not achieved by either approach, which can be attributed to the limitations of the utilized material model formulation.

(7) $$Difference=\frac{x_{1}-x_{2}}{\left(x_{1}+x_{2}\right)/2}\cdot 100$$

Table 3 displays the results of SM for each experiment and confirms the improvement of GA compared to the MF method. The biggest difference in SM can be observed with creep 1 (Figure 8b) of 0.1738 in favor of the GA. The final difference in the fitness score between the two methods illustrates a 40% overall improvement of the GA. The GA performed best with two-point crossover, a population size 150, and a mutation probability 0.1. The GA demonstrated its optimal performance with the use of a two-point crossover, a population size of 150, and a mutation probability of 0.1. The comparative charts of all the experiments with the solutions obtained from both the GA and MF methods can be found in Appendix A.

The optimization time can be improved by increasing the computing power, although this improvement will eventually be limited by the number of available Abaqus licenses. The simulation time required for the GA is another limiting factor of its usability. The complexity of the simulation model, the available computing power, and the availability of Abaqus licenses must all be considered when implementing the GA for material parameter optimization within industrial settings.

A significant portion of the simulation time is consumed by Abaqus performing sanity checks and validating the license. An open-source finite element method (FEM) solver, such as Calculix [26], that supports the desired material model can improve the optimization time and relieve the GA from the constraint of Abaqus licenses. It is based on the same input file structure as Abaqus, which means that the file structure can remain the same.

The GA is faster and more automated than the MF method, allowing the algorithm to run overnight and produce results for experts to review the following morning. The inputs to the algorithm are normalized experimental results and rough parameter ranges, making it readily deployable for new material parameter identification tasks. The results of the parameter study conducted in this work can be used as starting parameters for future tasks. By coding the algorithm in Python, it is made future-proof and flexible due to its open-source nature, while also reducing the final cost as there is no need for commercial software. The parallelization of the fitness function was a crucial factor in optimizing the algorithm’s performance and maximizing utilization of available hardware. The GA found an optimum in 37 iteration that took 18 h and 30 min.

The Pygad library used in this work employs bit-focused operators, which can be inefficient when dealing with real-valued genes. This article demonstrates that binary operators can be effective, but implementing operators better suited for real-valued genes can further improve the required optimization time. These operators are described in an article by R. Patel and M. M. Raghuwanshi [27].

## 4. Conclusions

What is known about the material is how it behaves from experimental tests. The objective of this work was to use FEM simulations to replicate these experiments and identify the material parameters by comparing the simulation results with actual experimental data. This article presents the development and initial parameter study of the GA used to perform this optimization task. Based on experimental data, FEM simulations, and specified parameter ranges, the GA is able to determine an optimal set of parameters. An analysis of the GA’s performance shows that the population size is the most influential parameter, with a population size of 150 yielding the best results. The mutation probability also plays a crucial role in the algorithm’s ability to explore the parameter space, and a value of 0.1 is found to be optimal. Crossover operators show negligible differences with each other. The comparison between the GA’s best solution and the current trial and error method used by AVL shows a 40% improvement in the fitness score. Scoring each individual depends on running Abaqus simulations which take time, computing power, and Abaqus licenses to run. Those are all limited resources, and a balance between them must be found for successful use of the GA. The development of the GA in this study was carried out within an industry environment, which presents additional constraints but also requires a flexible and efficient algorithm. The GA is coded in Python making it easy to modify or upgrade in the future. A parallelization of the fitness function managed to best utilize the available hardware and deliver results in 18.5 h. It offers a high degree of automation meaning the expert does not need to oversee the optimization.

## Figures and Tables

**Table 1:** Parameter limits and step sizes.

Parameter	Lower Limit	Upper Limit	Step Size
$R_{e}$	0.1	200	1
$C_{1}$	2000	20,000	100
$\gamma_{1}$	100	1000	4.5
$C_{2}$	0.1	2000	10
$\gamma_{2}$	0.1	1000	5
$\xi_{1}$	0.001	0.2	0.001
$R_{1}$	0.1	5	0.025
$m_{1}$	0.1	5	0.025
${\dot{\varepsilon }}_{0}$	0.1	15	0.075
K	100	1000	4.5
n	0.1	10	0.05

**Table 2:** Example of a chromosome with real values for genes.

$R_{e}$	$C_{1}$	$\gamma_{1}$	$C_{2}$	$\gamma_{2}$	$\xi_{1}$	$R_{1}$	$m_{1}$	${\dot{\varepsilon }}_{0}$	K	n
100	10,000	100	1000	100	0.1	1	1	10	100	1

**Table 3:** SM for parameters from the MF method and for the optimum found by GA are gathered in this table. In the last row, the calculated fitness score (Equation (6)) is presented. The difference is calculated by Equation (7) and is positive when it shows improvement of the GA.

Experiment	MF	GA	Difference [%]
LCF 1	0.0524	0.0335	44
LCF 2	0.1021	0.0817	22
LCF 3	0.0609	0.0440	32
LCF 4	0.0753	0.0706	6
LCF 5	0.0641	0.0509	23
LCF 6	0.0570	0.0392	37
LCF 7	0.0310	0.0279	11
Creep 1	0.1789	0.0051	189
Creep 2	0.1045	0.1040	0
Creep 3	0.1239	0.0979	23
Creep 4	0.0570	0.0258	75
Tensile	0.0446	0.0509	−13
Fitness score	1.05	1.58	40

**Figure A1:**
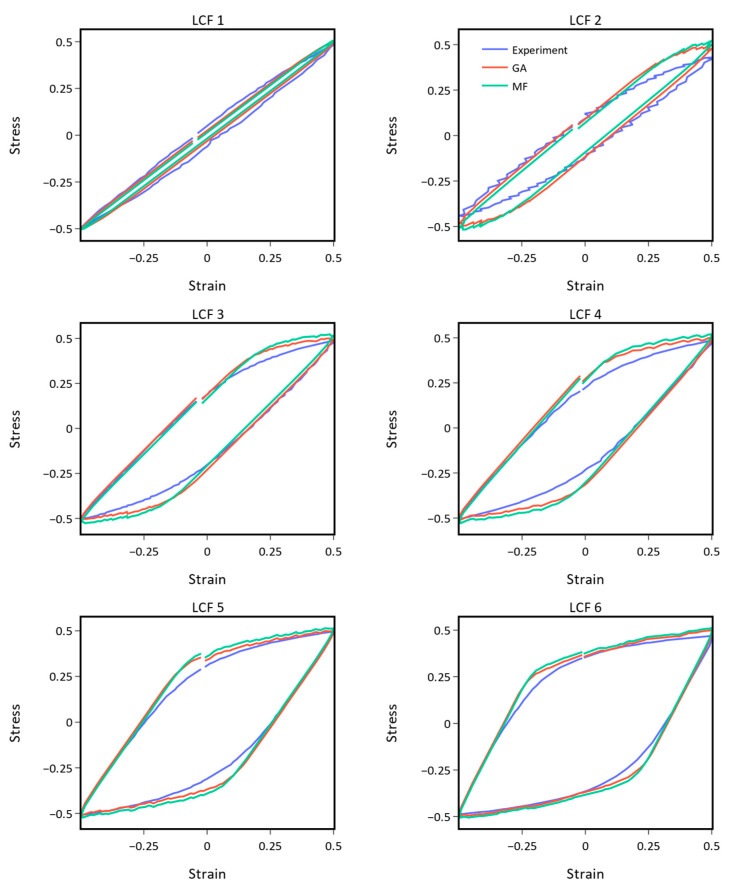
GA and MF methods compared to the experimental curves LCF 1–6.

**Figure A2:**
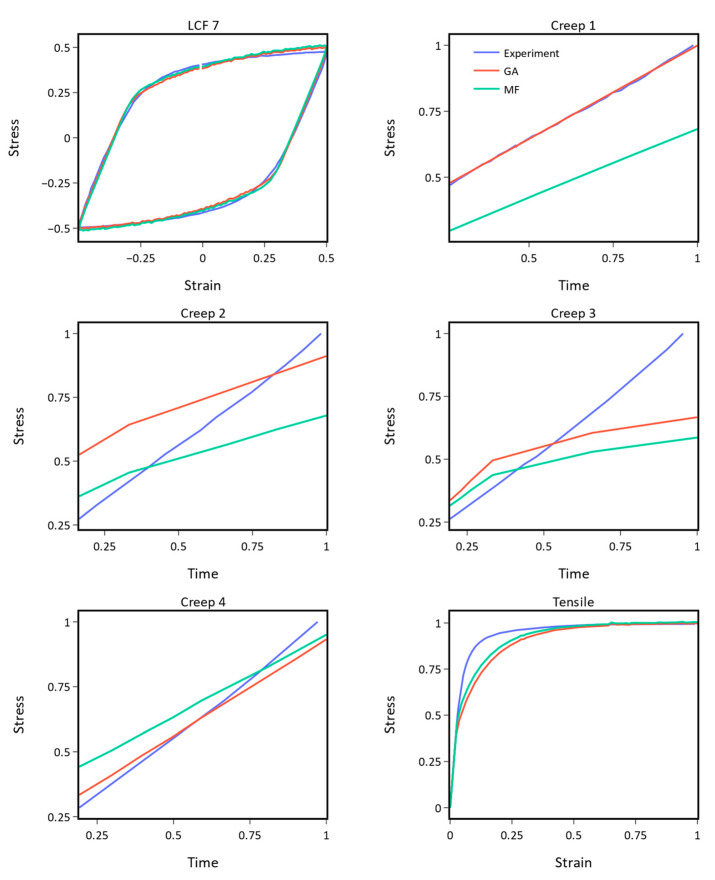
GA and MF methods compared to the experimental curves LCF 7, creep 1–4, and tensile.

## Data Availability

Not applicable.

## Appendix A

Figures for all 12 experiments, together with simulation results from the GA and MF methods.
